# Targeting climate adaptation to safeguard and advance the Sustainable Development Goals

**DOI:** 10.1038/s41467-022-31202-w

**Published:** 2022-06-23

**Authors:** Lena I. Fuldauer, Scott Thacker, Robyn A. Haggis, Francesco Fuso-Nerini, Robert J. Nicholls, Jim W. Hall

**Affiliations:** 1 Environmental Change Institute, University of Oxford, South Parks Road, Oxford, OX1 3QY UK; 2 KTH Climate Action Centre & KTH Division of Energy Systems, KTH Royal Institute of Technology, SE-100 44 Stockholm, Sweden; 3 Tyndall Centre for Climate Change Research, University of East Anglia, Norwich, NR4 7TJ UK

**Keywords:** Climate-change impacts, Climate-change adaptation, Sustainability, Natural hazards, Climate-change impacts

## Abstract

The international community has committed to achieve 169 Sustainable Development Goal (SDG) targets by 2030 and to enhance climate adaptation under the Paris Agreement. Despite the potential for synergies, aligning SDG and climate adaptation efforts is inhibited by an inadequate understanding of the complex relationship between SDG targets and adaptation to impacts of climate change. Here we propose a framework to conceptualise how ecosystems and socio-economic sectors mediate this relationship, which provides a more nuanced understanding of the impacts of climate change on all 169 SDG targets. Global application of the framework reveals that adaptation of wetlands, rivers, cropland, construction, water, electricity, and housing in the most vulnerable countries is required to safeguard achievement of 68% of SDG targets from near-term climate risk by 2030. We discuss how our framework can help align National Adaptation Plans with SDG targets, thus ensuring that adaptation advances, rather than detracts from, sustainable development.

## Abstract (web summary)

Without targeted climate adaptation, impacts of climate change threaten achievement of all 169 SDG targets. Fuldauer et al. provide an actionable framework to assess these impacts and help systematically align national adaptation plans with the SDGs.

## Introduction

In 2015, the world’s governments committed to achieve 169 targets under the global Sustainable Development Goals (SDGs) and to engage in climate adaptation planning under the Paris Agreement. Success in delivering on these two commitments will heavily depend on decision-makers to effectively plan and implement synergistic action between SDG target achievement and climate adaptation^1^. Yet, despite numerous calls to align national climate adaptation with sustainable development objectives^2^, these calls have not yet led to action^3^. To date, only four of twenty existing National Adaptation Plans (NAPs) mention the SDG targets (Supplementary Table 1).

Aligning SDG targets and climate adaptation is inhibited by siloed global and national governance. Another obstacle is the inadequate understanding of the complex relationship between SDG targets and climate adaptation at a useful scale to inform decision-making in practice. Currently, most national governments organise and implement adaptation plans at the sector scale (such as infrastructure, healthcare, ecosystems, etc.) (Supplementary Table 1). Yet, the research community has focused on investigating the relationship between the 169 SDG targets, the impacts of climate change, and climate adaptation at a broad scale^4^ and has not yet provided an actionable framework to systematically understand the role of sectors of the economy, society and the environment in mediating this relationship. Without a systematic understanding of how sectors mediate between SDG targets, impacts of climate change and adaptation, it is not possible to systematically align national adaptation plans with SDG targets or to account for the indirect and interdependent sectoral effects of how SDG targets are affected by various impacts of climate change. Yet, it is these indirect and interdependent cascading effects on SDG targets^5^ that are likely to lead to the most far-reaching risks^6^, and, inversely, allow for the greatest SDG benefits from adaptation.

In this paper, we aim to address these gaps by proposing and globally applying a sector-scale framework for targeting adaptation to safeguard and advance SDG targets. The framework builds on previous studies that mapped: (a) influences (also referred to as interlinkages in the literature) between specific sectors and specific SDG targets^7–13^, (b) influences between specific climatic impact-drivers, such as floods or chronic warming, and sectors^14–17^ and (c) interdependencies across SDG targets^18–22^. Whilst these previous studies have provided a conceptual basis for targeting sector-scale action in practice^23,24^, they share a number of limitations that inhibit informing national adaptation plans in alignment with sustainable development: (a) they have separated the SDG and climate adaptation field; (b) they have focussed on a selected set of SDGs, sectors, or climatic impact-drivers, and c) they have not accounted for interdependent sectoral effects on SDG targets.

**Fig. 1:**
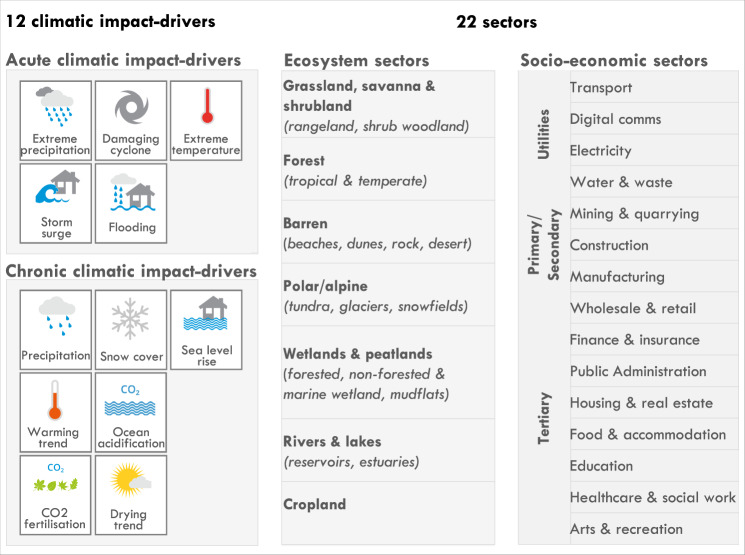
Summary of climatic impact-drivers and ecosystem and socio-economic sectors considered in this paper. The sector classification is used to simplify presentation and discussion and does not represent any hierarchy of sectors. We acknowledge the grey boundaries between sectors, and have hence performed the related analysis at the service-scale, i.e. based on the services that sectors provide (see “Methods”).

Our proposed framework integrates and expands upon past studies to conceptualise the complex bi-directional influences between all 169 SDG targets, 12 different acute (extreme) and chronic (slow-onset) climatic impact-drivers, and a holistic set of 22 ecosystems and socio-economic sectors (Fig. 1). By applying our framework globally, we show that all 169 SDG targets are threatened by near-term sectoral risk from climate change. To reduce these risks, adaptation of ecosystems can help safeguard and advance 62% of all 169 SDG targets, adaptation of utility infrastructure sectors can help safeguard and advance 81% of targets, adaptation of primary/secondary sectors can help safeguard and advance 40% of targets, and adaptation of tertiary sectors can help safeguard and advance all SDG targets if planned and governed well. Integrating ecosystems in climate adaptation will be essential to complement and substitute socio-economic sectors in achieving targets across 13 of 17 SDGs in the face of near-term sectoral risks.

## Results

### A framework for adaptation to safeguard and advance SDGs

**Fig. 2:**
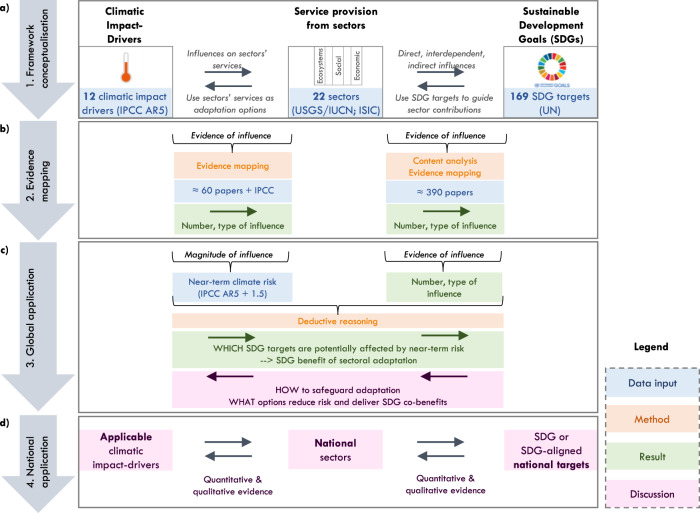
Conceptualisation and application of our proposed framework for targeting adaptation, which focuses on how sectors mediate between climatic impact-drivers and SDG target achievement. **a** Framework conceptualisation. **b** Evidence mapping based on potential influences. **c** Global application based on IPCC 5th Assessment Report initial global influences. **d** Considerations for national application of our framework. Icon images courtesy of United Nations.

Our proposed framework conceptualises how sectors mediate between climatic impact-drivers and SDG target achievement (Fig. 2a). It focuses on service provision from a holistic set of 22 ecosystems and socio-economic sectors that represent the three sustainable development pillars: environmental, social and economic^25,26^, and are based on international classifications^27–29^.

Using content analyses and evidence mapping, we populate our proposed framework with potential influences from sectors to SDGs, and climatic impact-drivers to sectors (Fig. 2b, rightward arrows). We first identified and mapped evidence of the influences between each sector and SDG target, differentiating by direct, indirect, and interdependent sector-SDG influences (for definitions, see Supplementary Table 2). Second, we mapped evidence of the influences between each climatic impact-driver and each sector, differentiating by climate-sector influences on a sector’s supply: (a) land/natural resources (e.g. affecting natural biodiversity), (b) physical capital (e.g. affecting factories/offices), (c) labour (e.g. affecting workers or productivity) and d) demand (e.g. affecting electricity demand). We refer readers to the methods section and to Supplementary Data Tabs 2.1–3.2 for a record of all influences, evidence, and word strings used.

We demonstrate how it is possible to build upon these potential influences with the best available global data on high near-term sectoral risk, which exemplifies one potential forward application of our framework (Fig. 2c). Near-term sectoral risk refers to the probability and magnitude of climatic impact-drivers affecting sectors by the 2030 s (aligned with the SDG timeline) and is defined based on the Intergovernmental Panel on Climate Change Fifth Assessment Report by Working Group II (IPCC AR5)^16^. Based on deductive reasoning, we link results from near-term sectoral climate risk to sector influences on SDG targets, which allows quantifying how many SDG targets are potentially affected by global near-term sectoral risk (see Supplementary Data Tab 4). We discuss how our analysis of potential and global influences can help work backwards from the SDGs to ensure adaptation safeguards and advance SDG targets and reduces sectoral risk.

National governments typically tailor adaptation plans according to their national circumstances and interpret the SDGs and Paris Agreement based on their level of development. We show how applications of our framework that consider nations’ differences in resources and geography provide practical guidance to align national adaptation planning with SDG targets and help leave no one behind (Fig. 2d).

### Influences among sectors and SDG targets

**Fig. 3:**
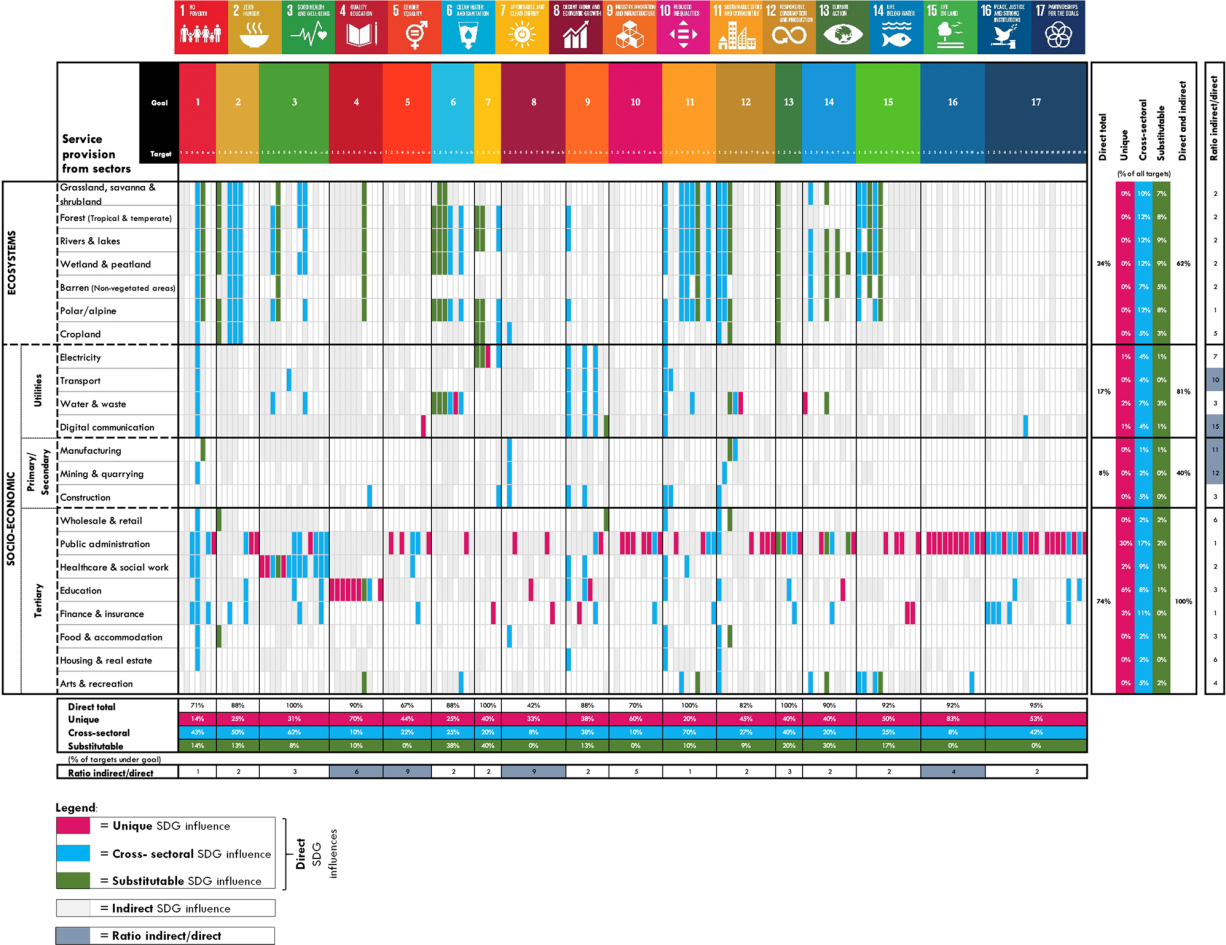
Ecosystems and socio-economic sectors influence SDG targets. Each rectangle represents one SDG target: magenta shading denotes a unique direct influence; blue a cross-sectoral influence; and green a substitutable influence for achieving the SDG target. Grey indicates an indirect influence, where there is published evidence that improving the quality/quantity of the sector’s services can help achieve the SDG target. White shading indicates the absence of identified evidence. Evidence is reported in Supplementary Data Tab 3.1. Icon images courtesy of United Nations.

Ecosystems and socio-economic sectors can directly influence SDG targets, based on the concept that these sectors provide services critical for development^7,10,30,31^. This first step of identifying potential influences involved content analyses of whether an SDG target is directly described in terms of the services a sector provides (see worked example in Supplementary Table 3 and see Supplementary Data Tab 2.1 for a detailed overview of the services provided by each sector). Natural or semi-natural ecosystem sectors include grasslands, savannas & shrublands, forests, rivers & lakes, wetlands & peatlands, barren, polar/alpine and croplands. Depending on their context, these ecosystems provide regulating (flood protection or carbon sequestration), provisioning (food, water, energy or medicines), supporting (habitat) and cultural services (heritage, recreational) that are critical for sustainable development^7,32^. Based on the services they provide, we find that all ecosystems directly influence 24% of all 169 SDG targets (Fig. 3, direct total). Beyond targets under SDG14 (life below water) and SDG15 (life on land) that explicitly mention ecosystems, 11 additional goals are described in terms of the services that ecosystems provide.

Socio-economic sectors, which include the three categories of utilities, primary/secondary, and tertiary sectors (see Fig. 1), also provide services that are critical for achieving SDG targets. Utility sectors (electricity, transport, water) provide infrastructure services, such as energy or mobility, that can directly influence 17% of SDG targets. Primary/secondary sectors (manufacturing, mining, construction) provide processing or installation services that we find can directly influence 8% of targets. Tertiary sectors (public administration, education, healthcare, amongst others) provide critical governance or educational services that can directly influence 74% of targets (Fig. 3, direct total). Compared to any other sector, the tertiary public administration sector directly influences most targets through the governance services it provides (50% of SDG targets). Identifying the SDG targets directly influenced by a sector’s services enables researchers and decision-makers to relate policy action on improving service provision directly to SDG target achievement.

We distinguish ecosystems or socio-economic sectors for practical reasons, acknowledging that multiple sectors act interdependently to provide services. For example, water services can be provided both by rivers & lakes and by utilities; ecosystem services such as flood protection can complement or substitute physical infrastructure services; and socio-economic governance services enable equitable ecosystems management. In addition, cultural services permeate through and across all sectors^33^. We consider interdependencies in direct SDG influences by accounting for whether sectors provide unique, cross-sectoral or substitutable contributions to SDG target achievement (Fig. 3). Whilst 43% of SDG targets are described in terms of a single sector’s service only (unique influence), 33% of targets mention multiple sectors’ services (cross-sectoral influence) and 11% are described in terms of a sector’s service that can be substituted by a different sector (substitutable influence). Identifying these types of interdependencies for each SDG target allows decision-makers to understand whether SDG target achievement is solely dependent on action within a single sector (unique influence) or whether target achievement can benefit from action within more than one sector (cross-sectoral; substitutable influence). In this context, our finding that targets across a total of 13 goals are directly described in terms of services from both ecosystems and socio-economic sectors reveals insight into the essential role ecosystems play in directly complementing and substituting socio-economic sectors in efforts towards sustainable development.

Ecosystems and socio-economic sectors can also indirectly influence SDG targets. Indirect SDG influences are defined as cases whereby a sector’s service is not directly mentioned in the target’s description, but for which published evidence indicates SDG target links (see worked example in Supplementary Table 3). For example, we find evidence that ecosystem services provide economic productivity and decent work benefits in relation to SDG8 (decent work)^31^. We identify on average five times more indirect SDG influences than direct ones (Fig. 3, grey shading). This ratio between indirect/direct SDG influences is highest for targets under SDG8 (decent work), SDG5 (gender equality), SDG4 (education) and SDG16 (peace). To ensure a decent work environment, a gender-equal world, quality education and peaceful living therefore does not directly emerge from scaling up sector’s services. It requires attention to the more hidden, indirect influences. Similarly, we find that the ratio between indirect/direct SDG influences is highest for the digital communications, mining, manufacturing, and transport sector. Achieving sustainable development benefits in these sectors needs explicit focus on indirect influences in order to maximise the full potential of sectoral investments for sustainable development outcomes.

### Influences among climatic impact-drivers and sectors

**Fig. 4:**
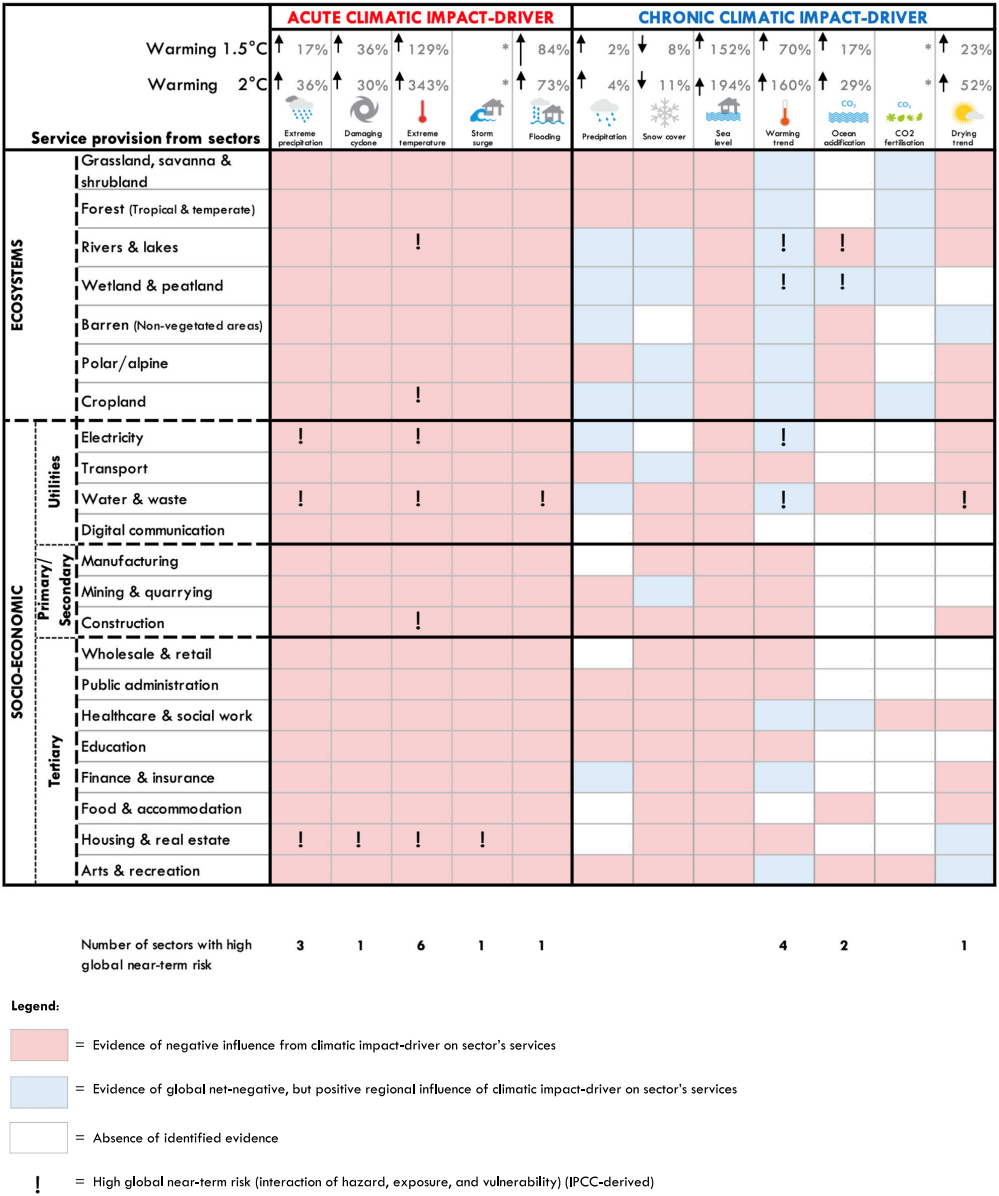
Ecosystems and socio-economic sectors influenced by acute and chronic climatic impact-drivers. Red shading denotes evidence of a negative effect, blue shading highlights a potential positive regional effect of a climatic impact-driver on a sector. White shading indicates the absence of identified evidence. Percentages for climatic impact-drivers signify changes in frequency under a 1.5 and 2 °C scenario (see Supplementary Data Tab 3.3), whereby the symbol * suggests that no quantified evidence was identified. The symbol ** denotes the grey boundaries on whether a drying trend and droughts are classified as chronic or acute climatic impact-drivers (see Supplementary Data Tab 2.1 for definition and justification). Exclamation marks represent high near-term risk based on IPCC AR5 TS.4^16^.

There is ample evidence of the influences between climatic impact-drivers and sectors (see worked example in Supplementary Table 4). Our evidence review reveals that acute climatic impact-drivers can threaten all 22 considered sectors through impacts on supply of, or demand for, sectors’ services (Fig. 4, red shading). Chronic climatic impact-drivers predominantly affect sectors negatively via impacts on land/natural resources, with some regional positive effects (Fig. 4, red and blue shading). For example, chronic warming is projected to reduce agricultural yields globally, but may increase yields in northeast China and the UK^34^.

Yet, the risk emerging from these climatic impact-drivers is highly context-dependent. It is largely based on the magnitude and frequency of the hazard (a climatic impact-driver with negative consequences) within a certain area, the exposure of sectors to the hazard, and the vulnerability of the sector to continue to provide its services when exposed to a hazard (a sector’s sensitivity) as well as the vulnerability of the population using the sector’s service (adaptive capacity) (see Supplementary Table 2 for definitions). Sectors where service provision is already poor, declining or endangered from other stressors are likely to be more vulnerable to additional impacts of climate change. Similarly, poorer societies are less capable to recover from climate-induced losses by means of diversification of incomes, amongst other factors^35^. Acknowledging the difficulty and complexities with any global evidence of climate-sector influences, we apply IPCC AR5s near-term sectoral risk ranking^16^. We find that all acute and three chronic climatic impact-drivers (the warming trend, ocean acidification and the drying trend) cause highest near-term risk to six sectors and the services these provide. These climate-sensitive sectors include rivers & lakes, wetland & peatland, cropland, electricity, water & waste, construction and housing & real estate.

### SDG targets influenced by impacts of climate change

**Fig. 5:**
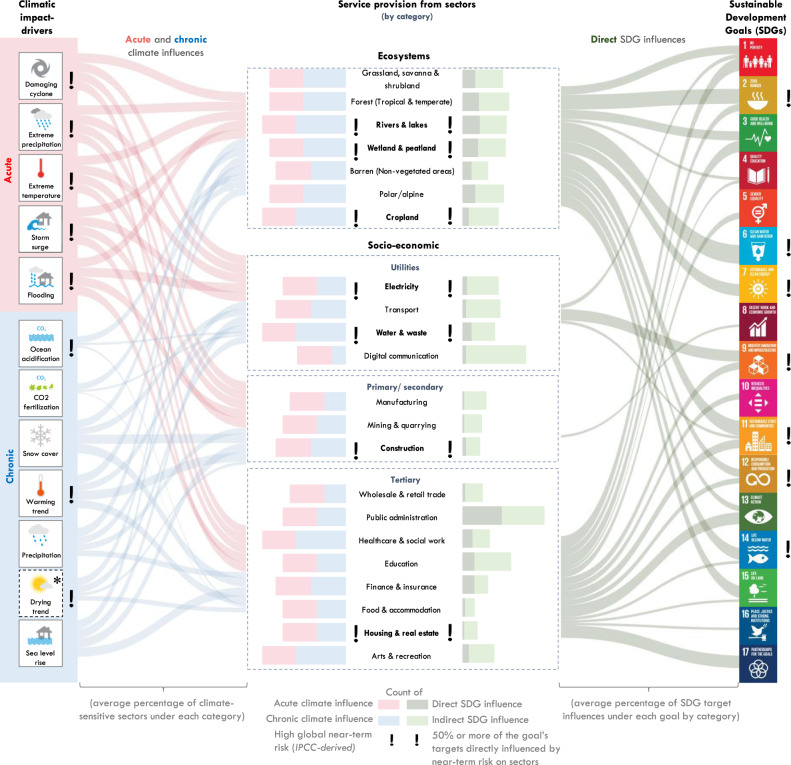
Results of the global application of our framework, showing sectoral risk from climatic impact-drivers on SDG target achievement. From left to right: Percentage of sectors under each category (ecosystems, utilities, primary/secondary, tertiary) influenced by acute and chronic climatic impact-drivers (red and blue Sankey lines); quantity of influences between acute and chronic climatic impact-drivers (red and blue bars) and sectors; quantity of direct and indirect influences between sectors and SDG targets (dark and light green bars); and the percentage of SDG targets under each goal directly influenced (green Sankey lines) by each category. Exclamation marks denote high global near-term sectoral risk. The symbol * denotes the grey boundaries on whether a drying trend and droughts are classified as chronic or acute climatic impact-drivers. Icon images courtesy of United Nations.

Based on deductive reasoning, we integrate: (i) sector-SDG influences and (ii) climate-sector influences. This allows us to identify that achievement of 146 of 169 SDG targets (86%) can potentially be directly undermined by any acute climatic impact-driver (Fig. 5). Chronic climatic impact-drivers, which have either negative or regionally positive effects, can threaten 37% more SDG targets than they can support through opportunities. Combining direct and indirect SDG influences, the achievement of all 169 SDG targets is potentially threatened by acute or chronic climatic impact-drivers. These results demonstrate the value of considering sectors as mediators between SDG targets and impacts of climate change: adopting a sector-scale approach provides a more nuanced understanding of the impacts of climate change on the SDGs as compared with recent literature in the field^4^.

An understanding of the magnitude of impacts of climate change on SDG targets in turn provides insight into the SDG benefits of adapting climate-sensitive sectors. When planned and governed well, adapted ecosystems can help safeguard 62% of all 169 SDG targets; adapted utility infrastructure sectors can help safeguard 81% of targets; adapted primary/secondary sectors can help safeguard 40% of targets; and adapted tertiary sectors can help safeguard all SDG targets. Notably, 21% of potentially climate-sensitive targets require adaptation across ecosystems and socio-economic sectors.

Based on IPCC AR5’s global data on near-term sectoral risk, the seven sectors at highest near-term risk can directly hamper the achievement of 36% of SDG targets (Fig. 5, exclamation marks). Especially affected is the achievement of SDG2 (end hunger), SDG6 (clean water), SDG7 (energy), SDG9 (innovation and infrastructure), SDG11 (sustainable cities), SDG12 (responsible consumption and production) and SDG14 (life underwater), where a minimum of half of the targets under the respective SDG are directly influenced by one or more of the seven sectors (see Supplementary Data Tab 4 for target-level results). When considering both direct and indirect sector-SDG influences, high near-term risk from climate change on the seven sectors can affect 68% of SDG targets across all 17 goals. Focussing adaptation on these seven sectors this decade is therefore critical to safeguard achievement of 68% of SDG targets by 2030.

## Discussion

### Tailor adaptation to safeguard SDG targets and advance SDG target co-benefits

Our understanding of types of influences can help work backwards from the SDGs to specify: (1) where and how to adapt sectors to safeguard SDG target achievement, and (2) what type of sectoral adaptation option might reduce climate risk and advance SDG target co-benefits, which we describe in the context of our global near-term sectoral risk findings.

In tailoring adaptation to safeguard SDG targets, we apply our findings of how sectors can influence targets (unique, cross-sectoral, substitutable, indirect). For SDG targets which are directly influenced by a single climate-sensitive sector, adaptation can focus uniquely on that sector. In the absence of information on where risk on sectors is highest, adaptation of climate-sensitive public administration facilities, which are responsible for implementing just policy, can uniquely safeguard most SDG targets (30%) as compared to other sectors (Fig. 3, magenta shading).

For SDG targets that are influenced by different climate-sensitive sectors, cross-sectoral adaptation is needed. Compared to other sectors, we find that adaptation of climate-sensitive public administration facilities and ecosystems can safeguard most SDG targets through cross-sectoral contributions (17 and 12% of SDG targets, respectively) where each service provides an independent contribution to target achievement (Fig. 3, turquoise shading).

For SDG targets influenced by climate-sensitive sectors that provide substitutable functions, decision-makers have more options in their choice of where to adapt. We find that protecting or enhancing ecosystems—including rivers & lakes, wetlands & peatlands, and forests—can safeguard 9%, 9%, and 8% of SDG targets, respectively, through substituting services provided by climate-sensitive socio-economic sectors (Fig. 3, green shading).

In tailoring adaptation to reduce risk and to advance SDG target co-benefits, we differentiate by the three components of risk: hazard, exposure, and vulnerability (see Supplementary Fig. 1 for an example). Firstly, decision-makers might focus adaptation on geographic areas projected to experience more frequent/severe hazards. Hazard-based adaptation options often involve scaling up ecosystems’ adaptation services, such as restoring wetlands that reduce flood severity^36^. Our influence findings show that ecosystems’ adaptation services, especially when implemented in a manner that maximises potential indirect influences, have the potential to advance up to 104 SDG target co-benefits (see Supplementary Data Tab 3.1). As hazard-based options often focus on geographic areas, they can help protect more than one sector against hazards.

Secondly, decision-makers might tailor adaptation to address the exposure of a sector (land/resources, physical capital, labour) or the demand for its services (Supplementary Data Tab 3.2). For example, we identified evidence that outdoor-working agricultural labour (agricultural workers) is mainly exposed to extreme temperature, whilst agricultural physical capital (machinery, factory) is mainly exposed to floods. Therefore, decision-makers might choose working hour policies as an adaptation option to reduce exposure of labour, and land-use policies to reduce exposure of physical capital. Given that exposure-based adaptation options focus on location adjustments, substituting service provision away from exposed sectors may be a suitable adaptation option to reduce exposure.

Thirdly, decision-makers may tailor adaptation based on which populations or sectors are most vulnerable to hazard exposure. For example, poor agricultural workers who are already working under insecure arrangements or with little access to diversified resources have less capacity to adapt^16^, and agricultural factories that are built without resilient designs may be more sensitive to intense floods. As vulnerability-based adaptation options often focus on ensuring continued service provision to already vulnerable populations or climate-sensitive sectors in the face of hazard exposure, vulnerability-based adaptation may be suitable especially in cases where sector’s service provision cannot be substituted and/or hazard exposure cannot be reduced. Considering the potential hidden indirect SDG influences of scaling up governance or essential services related to vulnerability-based options can help maximise SDG co-benefits. In practice, hazard, exposure and vulnerability-based options might often be implemented together, and will likely be most effective for near-term hazards of high probability and relatively low impact^37^.

In light of our global near-term sectoral risk findings, we identify that safeguarding those SDG targets affected by multiple climate-sensitive sectors—especially under SDG2 (end hunger), SDG9 (innovation and infrastructure), SDG11 (sustainable cities), and SDG12 (responsible consumption and production)—necessitates cross-sectoral adaptation to multiple climatic impact-drivers. Nature-based Solutions (NbS), which include scaling up ecosystem protection or restoration in a specific geographic area (e.g. greening)^38^, could provide a valuable adaptation option to protect these multiple climate-sensitive sectors against various climatic impact-drivers (hazard-based). If they respect cultural and ecological rights and support biodiversity^38^, NbS options can—in addition to their adaptation services—also provide other cultural and regulating services that advance SDG co-benefits.

To globally protect achievement of the near-term climate-sensitive targets under SDG6 (clean water) and SDG7 (energy) with many substitutable influences, decision-makers may focus adaptation on sectors that provide multiple services in order to maximise SDG co-benefits. For example, achievement of SDG6 (water) could be protected by prioritising river & lake or wetland & peatland ecosystems less exposed by chronic warming to substitute for, or complement, climate-sensitive dams (exposure-based). Such substitutive or complementary action can not only safeguard the ecosystems regulating and cultural services. It also builds both social capital and adaptive capacity through community-based ecosystem management^39^.

Some SDG targets, especially under SDG11 (sustainable cities) and SDG14 (life under water), are influenced by climate-sensitive sectors that provide globally non-substitutable services towards wellbeing outcomes^40,41^. For example, rivers & lakes, forests and wetland & peatland ecosystems provide regulating air purification as well as natural and cultural heritage services that are globally non-substitutable in their contribution to targets under SDG11 and SDG14. These regulating and cultural services are already threatened by other non-climatic stressors. These non-climatic stressors include, amongst others, the declining contribution of ecosystem regulating and cultural services to the SDGs over time^42^. Therefore, to help safeguard SDG targets influenced by non-substitutable ecosystem services, management and/or conservation of such highly productive ecosystems is essential (vulnerability-based).

### Reflections and considerations in applying the conceptual framework

**Fig. 6:**
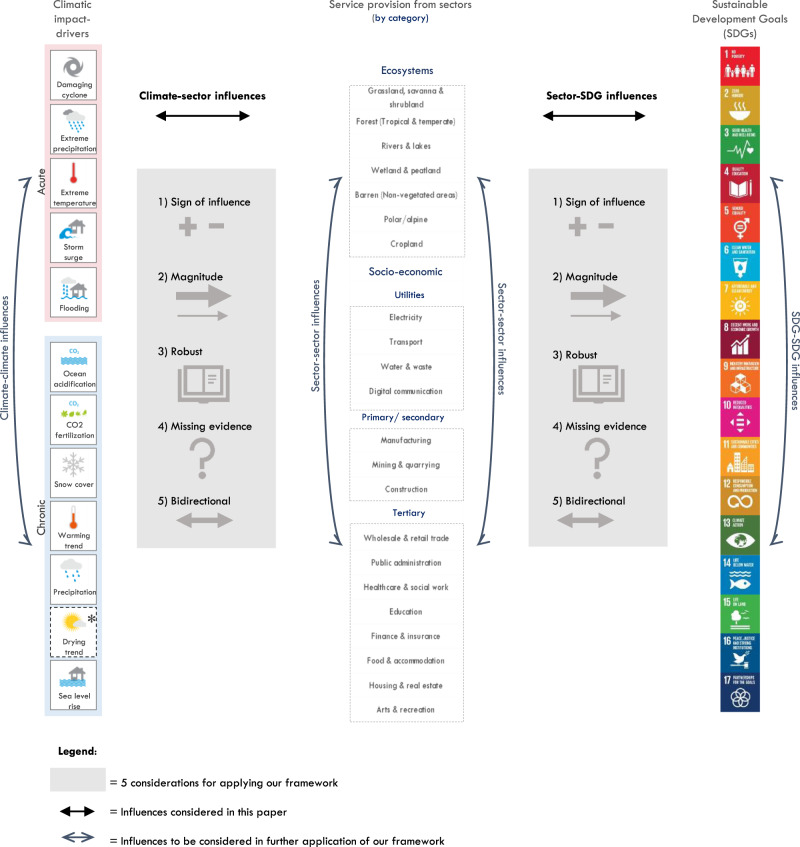
Five considerations in applying the proposed framework on influences amongst climatic impact-drivers, sectors and the SDG targets. Grey shading illustrates the five considerations. Bold black arrows depict influences considered in this paper, blue light arrows show future influences of climate–climate (compound events), sector–sector (sectoral interdependencies), and SDG–SDG influences (SDG interdependencies) to be considered in the national application of our framework. The aim of the application determines the relative importance of each consideration and whether a climate-first (from climate via sectors to SDG targets) or a development-first (from SDG targets via sectors to climate) approach is adopted. The symbol * denotes the grey boundaries on whether a drying trend and droughts are classified as chronic or acute climatic impact-drivers.

Adaptation decision-making is a complex and multifaceted challenge. Yet, governments and development organisations must urgently make decisions on adaptation that intersect with different dimensions of development^43^. To avoid maladaptive outcomes, decisions on adaptation should not only reduce risk, but also safeguard and advance sustainable development^44,45^. Although both research and real-work experience underscores the criticality of better understanding the relationships between SDG targets and climate adaptation to inform such synergistic decisions at the sector scale^46^, this understanding has been incomplete and inconsistent to date. Our proposed framework based on influences provides a starting point to navigate these complexities. Yet, its application must account for spatial and temporal dynamics and requires a number of considerations (see Fig. 6).

First, is the sector-SDG and climate-sector influence positive or negative? Negative sector-SDG influences might reveal opportunities to reverse trends and scale-up SDG contributions. Reversing negative trends can be realised by working backwards from potential indirect sector influences in a way that the SDG targets provide a framework to guide sustainable action. For example, negative influences from infrastructure sectors on ecosystems can be partly offset by embedding ecosystem considerations and NbS at the outset of any infrastructure project. Such design changes have the potential to maximise indirect influences of infrastructure services on SDG15 (life on land). Similarly, positive climate-sector influences, such as increased agricultural crop yields under warming trends, might be exploited in a way to scale-up sectoral service provision that benefits the poorest (SDG1 poverty).

Second, is the magnitude of the influence direction strong or weak? A strong sector-SDG influence (the SDG target is near full achievement based on a sector contribution) coupled with a strong climate-sector influence (sector is at high risk from climate change) suggests adaptation must be targeted to safeguard existing SDG progress. Conversely, a weak sector-SDG influence with a strong climate-sector influence (low baseline progress on SDG targets influenced by climate-sensitive sectors) suggest future SDG investments must be climate-resilient. An analysis of substitutable sectoral influences may help guide how to replace climate-sensitive with more resilient services to safeguard SDG target achievement.

Third, how robust is the influence, now and into the future? In other words, under what circumstances might the influence prove to be different to what is anticipated? Sectoral adaptation might be targeted to reduce climate risks and maximise SDG co-benefits across those influences with the highest robustness first (e.g. focusing on near-term sectoral risks attributable with high confidence), whilst creating iterative processes to monitor and assess less robust influences.

Fourth, where are gaps in our understanding of influences, including climate–climate (compound events^47^), sector–sector (cascading sectoral interdependencies^48–50^) and SDG–SDG (SDG interdependencies^19,51,52^) influences? Missing evidence for influences can help identify research gaps, but might also provide clues on existing sustainability or climate-resilience efforts or network effects that are working. Assessing self-reinforcing and cascading interdependent influences is critical to start to understand trade-offs and synergies amongst climate risks, sectors, and SDG targets, evidence of which is limited but emerging^17,47,53,54^.

Fifth, is the influence bi-directional? A sector can demonstrably influence SDG targets, whilst working backwards from an SDG vision might provide useful insights on non-existing influences and thereby guide future research. Similarly, climate change may influence a sector for which risk-reduction efforts may be infeasible. This is the case with some degraded ecosystems that have reached adaptation limits^55^, rendering risk-reduction options largely impossible.

The aim of the application will determine which of these considerations is particularly important, and how so. Climate-first application of our framework (from climate via sectors to SDG targets) can help researchers and decision-makers assess and quantify impacts of climate change on sectors and its interdependent influences on SDG targets, as demonstrated in a recent national-scale assessment in Saint Lucia^56,57^. Such climate-first applications make the SDG case for sectoral adaptation, transcending economic adaptation assessments.

Development-first application (from SDG targets via sectors to climate) can help ground climate adaptation in an SDG vision. By working backwards from national development targets, it is possible to evaluate the extent to which existing service provision across sectors already contributes to SDG targets and where sector adaptation provides the largest safeguarding gains, as demonstrated in a recent national-scale assessment in Ghana^58,59^. Similarly, development-first applications can help identify where and how future SDG investments require climate-resiliency considerations.

Sector-first applications (from sectors to SDG targets and climate) can support infrastructure planners or land-use managers to better tailor sector adaptation. This includes identifying where sectoral action can maximise positive SDG target influences, minimise negative ones, and reduce potential climate risks across different hazards. Opportunities for cross-sectoral coordination can be teased out, both in enhancing SDG target outcomes and in reducing risk.

### Towards coordinated global and national efforts

Whilst the global application of our proposed framework described in this paper made use of the best available global evidence on the sectoral risk magnitude across a range of climatic impact-drivers, it was hampered by a limited scope of sectors and missing evidence on the sign and magnitude of sector-SDG influences (see Limitations). Building on efforts such as the ISIMIP^60^ or the SDSN network^61^ and making use of machine learning^62^, centralised global databases that bring together siloed data in an updated manner are essential to build a more substantive and dynamic evidence base.

Yet, differences in geography and resources make it difficult and risky to apply generalised, global influences across highly diverse subnational contexts and countries. In some countries, application with national quantitative data on hazard scenarios, sectoral service information^14,43,64^, and quantified SDG linkages^23^ might be possible. Given the focus of this framework on a holistic set of internationally classified sectors, further integration with input-output models^64^ could enable better quantifications of the cascading effects across sectors in influencing sustainable development outcomes. Multi-criteria decision analysis can help simulate the reliability, multifunctionality^30^ and potential maladaptation consequences^65^ of nature-based solutions alongside engineering-based adaptation options^66^, including in the context of the SDG targets.

In more data- or resource-scarce countries, the framework might be used as a more structured process to aid participatory discussions on which hazard, sectors, and SDG linkages exist, resulting in qualitative scoring of adaptation priorities. Potential adaptation options can be discussed in multi-stakeholder workshops to identify where and how adaptation can advance synergistic, and reduce negative, SDG target outcomes.

Yet, unless the voices of local, marginalised and indigenous peoples are explicitly accounted for, any application of the proposed framework will fail in delivering sustainable development outcomes. The most successful applications of the framework will promote citizen buy-in from those already most left behind, account for issues of equity, fairness and justice^67^, and consider the non-substitutable value of ecosystems in multifunctional landscape approaches^68^. When such applications are also grounded in living data systems and use the best available science, they can play an important role in developing dynamic climate adaptation plans that leave no one behind.

### A call for scaling up sustainable climate adaptation

To date, calls for aligning climate adaptation with the SDGs have failed to deliver action^3^. Yet, we have demonstrated that without targeted climate adaptation action across sectors, the achievement of all SDG targets is being threatened by the impacts of climate change. As more nations around the globe revise their adaptation plans and commitments under the Paris Agreement, there is an urgent need for an actionable framework to help exhaustively align national adaptation plans with SDG targets. If planned and governed well, application of our proposed framework can help stir the action needed to ensure that adaptation is advancing, rather than detracting from, sustainable development.

## Methods

### Overview of three-step methodology

**Fig. 7:**
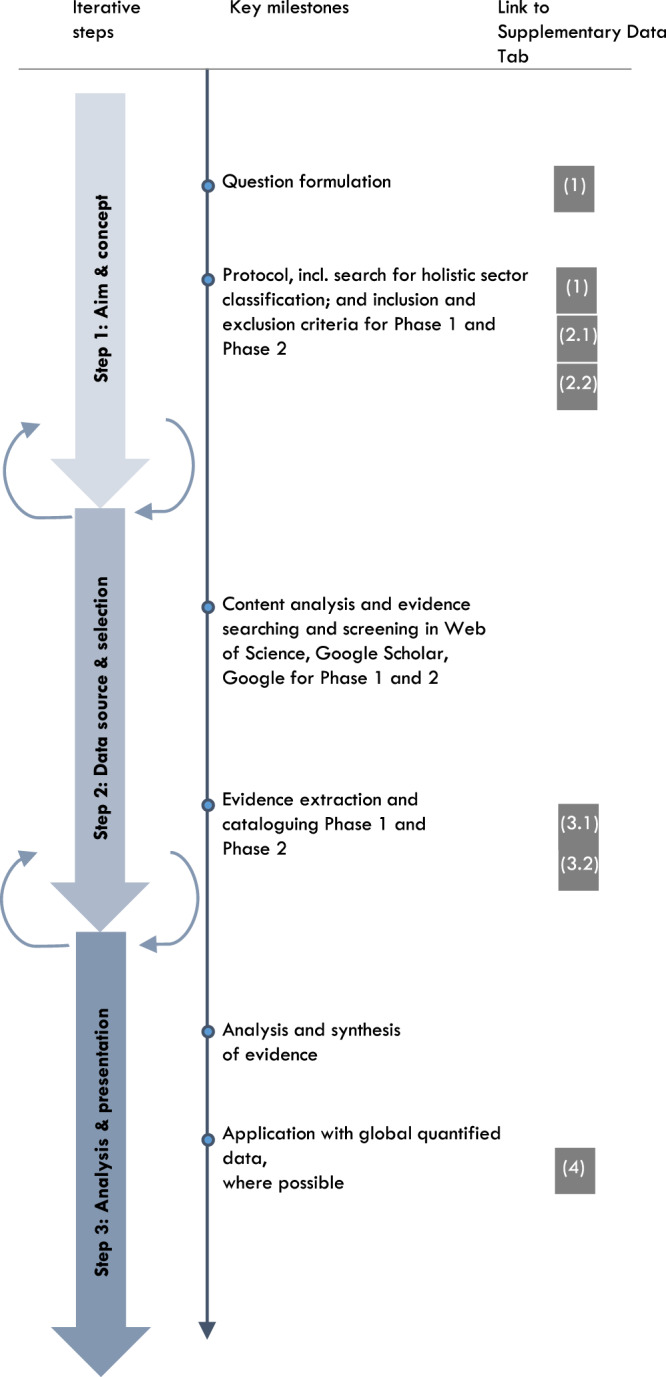
Overview of the method used to develop and apply our proposed framework for targeting adaptation to safeguard and advance the SDG targets. Left column introduces three main steps of the method, second column shows key milestones within each of the three main steps, third column illustrates the link to the Supplementary Data Tab.

The conceptualisation and application of this work followed a set of three best-practice steps to increase the methodological transparency and rigour in synthesising adaptation research as proposed by Berrang-Ford^69^ (see Supplementary Data Tab Contents for a detailed description of each step and its application to this research; see Fig. 7 for visualisation). The first step involved contextualising the research problem and identifying a conceptual approach to develop the framework. Search terms and inclusion and exclusion criteria were formulated to populate the framework with influences. The second step involved searching, screening, and cataloguing published evidence. This evidence was then characterised through descriptive statistics in the third step, which included mapping the quantity of sector-SDG and climate-sector influences for each sector. These steps were applied with IPCC-derived global data on near-term sectoral risk from 12 climatic impact-drivers and can be further applied at the national scale.

### Step 1: aim and concept

The aim of this research was to identify a framework for contextualising the complex relationship between the achievement of sustainable development targets and different impacts of climate change to provide a direct entry point for decision-makers to target climate adaptation for sustainable development outcomes. Acknowledging the complexities and ambiguities in conceptualising sustainable development and climate adaptation, we adopt normative definitions of the two concepts in the context of the global SDGs and the Paris Agreement (these normative definitions represent the best globally available compromise between the scientifically necessary and the politically possible response to address sustainable development and climate change, thereby reflecting a multiplicity of concerns and interests^46,70^). Previous literature has identified that providing direct entry points for decision-makers at different scales (global, national, public and private sector, academic modelling) requires the role of an intermediary^63^. We identified the following set of criteria for such an intermediary in the context of the SDGs and the Paris Agreement:

i. mentioned in nations’ sustainable development plans, Nationally Determined Contributions (NDCs), and/or National Adaptation Plans (NAPs),
ii. action within the intermediary can influence SDG target achievement and the adaptation component of the Paris Agreement (as an operator) and is influenced by the stimulus of climatic impact-drivers (as an exposure unit or receptor), following an existing framework on adaptation^71^,
iii. mappable, i.e. allows for a quantitative, GIS-based translation,
iv. globally applicable, i.e. consistent with international accounting standards, such as the System of Environmental Economic Accounting (SEEA) and global modelling standards to allow comparison across nations.

We used a holistic set of 22 ecosystems and socio-economic sectors as an intermediary, because nations’ development and climate adaptation plans are typically organised in terms of sectors and because sectors are both essential for achieving the SDGs and affected by climatic impact-drivers. Given the physical dimension of climatic impact-drivers, we based our choice of ecosystems and socio-economic sectors on the original land-cover/land-use classification categories by USGS^29^, which was developed using strict criteria to ensure mappability (i.e. spatial units are geographically exclusive and exhaustive). As the USGS classification was the first classification of land-cover/land-use, a range of global land-cover classifications build on it, making it globally applicable^28^. In addition, geospatial data for the terrestrial and freshwater categories is available at high resolution^28^. We updated the USGS classification categories according to the SEEA-based USGS categories of major ecosystem types^28^, which also aligns with the ecosystem types developed by the International Union for Conservation of Nature (IUCN) (see Supplementary Data Tab 1). We further disaggregated USGS’ classification of built-up land by socio-economic sectors, using the International Standard Industrial Classification (ISIC Rev 4^27^) of economic activities. ISIC is an internationally-used classification of socio-economic sectors that allows for the integration of open-source spatial data and that is consistent with international accounting standards.

Given that the value of a sector is determined by the services it provides over its life^32^, our analysis focused on the concept of sector’s services (including goods and resources)^7,10,30^. We explicitly catalogued the range of services provided by each of the 22 ecosystems and socio-economic sectors, based on international classifications where possible (see Supplementary Data Tabs 1 and 2.1). We identified a total of 35 different services provided by all ecosystem sectors, and a total of 32 different services provided by all socio-economic sectors. We grouped ecosystem sectors based on where they provide the same services, acknowledging that the quantity or quality of service provision may differ within the same sector (e.g. tropical forests provide much larger mitigative services compared to temperature forests, but both tropical and temperature forests are grouped under the forest sector category). For socio-economic sectors, we applied the services provided by each sector as stated in ISIC Rev 4. Recognising that the grouping of services into single entity sectors masks important differences between sectors and across different national accounts (see Limitations), it nevertheless provides a useful means by which to present and discuss findings that are transferable and can be expanded upon in national applications.

We populated our framework based on content analyses and evidence mapping of influences through two main phases (see Supplementary Data Tabs 2.1–3.2 for definitions, specific inclusion and exclusion criteria and catalogue of literature evidence).

Phase 1 included content analysis and evidence mapping for sector-SDG influences, differentiating by the type of influence. For each of the 169 SDG targets and each of the 22 sectors, we analysed and catalogued influences by answering the following questions:

1.1 *Direct SDG influences*: “Using the official UN wording of the SDG targets, is the SDG target directly described in terms of the services provided by the sector”? (search terms included the wording of SDG targets and the sector’s services as described in Supplementary Data Tabs 2.1 and 3.1)
  For example, target 11.6 “By 2030, reduce the adverse per capita environmental impact of cities, including by paying special attention to air quality and municipal and other waste management” is directly described in terms of the purification of air services provided by forests, and the waste management services provided by the water & waste sector.
1.2 *Interdependent SDG influences:* ‘For each SDG target directly described in terms of the services provided by a sector, how many sectors’ services are mentioned in the description of the SDG target?’
  If “1”, classify as *unique SDG influence*. A unique influence is identified when an SDG target is described solely in terms of one sector’s service, in other words, the sector provides an independent, singular contribution towards SDG target achievement. For example, target 16.3 “Promote the rule of law at the national and international levels” is uniquely influenced by (i.e. directly described in terms of only) the law enforcement services provided by the public administration sector. This function cannot be substituted by the services of another sector.
  If “2” or more, “does each sector provide a different service, in other words an independent contribution towards SDG target achievement?”
  If “Yes”, classify as *cross-sectoral SDG influence*. A cross-sectoral influence is identified when a sector’s service provides independent, cross-sectoral contributions towards the achievement of an SDG target. For example, target 11.4 “Strengthen efforts to protect and safeguard the world’s cultural and natural heritage” is described in terms of both cultural heritage services from the arts & recreation sector as well as natural heritage services from a range of ecosystem sectors, including the forest sector. Both services are needed to ensure target achievement.
  If “No”, classify as *substitutable SDG influence*. A substitutable influence is identified when sectors provide a service that can be substituted by another sector. In such a case, various sectors provide the same service towards SDG target achievement, presenting decision-makers with a choice of how to safeguard target achievement in the face of impacts of climate change. For example, target 6.1 “Achieve universal and equitable access to safe and affordable drinking water”, can be influenced by the water provision services directly abstracted from either rivers & lakes *or* from water & waste utilities.
1.3 *Indirect SDG influence:* “Is there published evidence that achievement of the SDG target can be indirectly influenced by the services provided by the sector?” (search terms included the wording of SDG targets and the sector or sector’s services, as described in Supplementary Data Tabs 2.1 and 3.1).
  An indirect influence is identified where the SDG target is not described specifically in terms of the service that a sector provides, but for which published evidence indicates that improving the quality or quantity of the service provided by a sector can enhance the achievement of the target, following the definition of Thacker et al.^10^. For example, target 5.2 “Eliminate all forms of violence against all women and girls […]” can be indirectly influenced by the healthcare & social work sector, as there is evidence that improving the quality of healthcare services, especially drug addiction services, can reduce violence^72^. Indirect influences include cases whereby there is published evidence that improvements in environmental management or fair service provision can support achievement of the target. It excludes cases of second-order interdependencies: for example, there is no indirect influence between the mining & quarrying sector and SDG target 11.1 (“Ensure access to housing”), because only a second-order influence could be identified: mining & quarrying supports the provision of minerals, which are then used in construction of housing shelter (see Supplementary Data Tab 3.1 for all inclusion and exclusion criteria).

Phase 2 included evidence mapping of climate-sector influences, differentiating by the type of influence. Thereby, we categorise 12 climatic impact-drivers, as defined by the IPCC AR5^16^, into acute (extreme) and chronic (slow-onset) climatic impact-drivers (Supplementary Data Tab 2.2). For each of the 12 climatic impact-drivers and each sector, we analysed and catalogued influences for the following questions:

2.1 *Climate-sector influence*: ‘Is there published evidence that the climatic impact-driver can negatively or positively influence the quantity or quality of the services provided by the sector via impacts on land/natural resources, physical capital, labour) or demand?’ (search strings included the wording of the climatic impact-drivers, various strings for supply and demand factors, and the sector’s services as described in Supplementary Data Tab 3.2).
  If “Negative”, encode as “−”, include the type of impact, and include confidence of the published evidence if available.
  If ‘Positive, encode as “+”, include the type of impact, and include confidence of the published evidence if available.

If reference was made to coastal infrastructure, any socio-economic sector was considered to be potentially affected. If the literature made reference to extreme events, the following climatic impact-drivers were included: extreme precipitation, damaging cyclone, extreme temperature, flooding, storm surge. Climate-sector influences exclude evidence in which climatic impact-drivers can result in economic market readjustments (e.g. increased prices due to shortage of supply following extreme impacts) and do not differentiate by geographic regions (see Supplementary Data Tab 3.1 for all inclusion and exclusion criteria).

### Step 2: data source and selection

The content analysis for the direct sector-SDG influences used the wording of the SDG targets and the sector and services of each sector (see search strings in Supplementary Data Tab 2.1). The search for scientific evidence for the indirect sector-SDG and the climate-sector influences was conducted in three different stages (in order of search): (1) Tier 1 journals and IPCC assessments and special reports (for climate-sector influences, including Global Warming of 1.5 °C^67^, AR5^16^, Climate Change and Land^73^), (2) other peer-reviewed articles and preprints, and (3) Grey literature (reports from international organisations, national and subnational agencies). The search for evidence was first performed through Web of Science, which was chosen given its high speed of inclusion of related articles and the inclusion of preprints. Second, a google scholar and google search was used to identify evidence from stage 3 (Grey literature). English was used for the evidence search, as it is the most employed language and considered as the international academic language^74^.

The evidence for each influence was screened against the predetermined definitions and the inclusion and exclusion criteria. In the case of ambiguous influences, these were reviewed and discussed amongst the author team until a consensus was reached, following the process adopted in previous studies^4,10^. One piece of evidence was considered sufficient to warrant an influence. All evidence was catalogued in Supplementary Data Tabs 3.1 and 3.2. The search for evidence aimed to gain a view on whether potential influences exist rather than a systematic review of all published evidence. We did not conduct a meta-review of the evidence to characterise the quality of the evidence, but hope to mitigate this aspect through our prioritised search in different stages and by embedding confidence intervals where available. We discussed how potential influences could be realised in practice with evidence on actual or projected influences (see the section “Reflection and considerations in applying the conceptual framework”).

### Step 3: analysis and presentation

The sector-SDG influence findings from the content analysis and evidence mapping were characterised through descriptive statistics, both at the sector and SDG target level. At the sector level, we summarised the quantity of sector-SDG influences in terms of absolute numbers and percentages of SDG targets (Fig. 3). At the SDG target level, we summarised the number of sectors that can potentially influence that SDG target as well as the type of influence (direct, indirect, interdependent). To provide useful implications for decision-making, we further summarised results through aggregating sector results at the category scale: ecosystems; utilities, primary/secondary (economic sectors); and tertiary sectors (social) (Fig. 2). For the sector-SDG influences, we did not assess the magnitude of influences, because such information was not available at the global scale across all ecosystems and socio-economic sectors, and is highly context-specific. Global indicator data to measure the magnitude of SDG target achievement exists^75^, but this is determined by the availability of data across all nations, does not capture the contribution of sectors as described in this paper, and does not allow accounting for indirect and interdependent influences.

For the climate-sector influences, we described the number of potential negative or positive influences from each climatic impact-driver on each sector (Fig. 4). We summarised how climatic impact-drivers influence each supply factor (land/natural resources, physical capital, labour) or demand for each sector (see Supplementary Data 3.2).

Unlike for the SDG influences, global data on the magnitude of influences from different climatic impact-drivers on sectors was available. We therefore applied data from IPCC AR5s key sectoral risk ranking (Table TS.4)^16^, the best globally available ranking of risk across 12 different climatic impact-drivers and across sectors. We used near-term sectoral risk to align with the 2030 SDG timeline. A sector was marked as being at high global near-term risk if IPCC AR5’s Table TS.4^16^ identifies the sector (or the sector’s services, as worded in Supplementary Data Tab 2.1) as being at high or very high risk of the specific climatic impact-drivers with current adaptation levels and high confidence (see Supplementary Data Tab 4 for results).

Based on deductive reasoning, we combined sector-SDG (Phase 1) and climate-sector influences (Phase 2) in order to compute how each SDG target can be affected by climatic impact-drivers via effects on sectors’ services (a climate-first application of the proposed framework). For example, if there is published evidence of a negative effect of chronic warming on cropland-based food production, we compute the number of SDG targets directly and indirectly influenced by cropland-based food production. We repeated this step with our IPCC-derived global data on the magnitude of near-term sectoral risk to identify the potential SDG targets influenced by those sectors/ services at near-term climate risk (see Supplementary Data Tab 4).

### Limitations and future work

#### Sector definition/scope

There are many ways that sectors and services can be classified, a main determinant of the proposed framework and influences presented in this paper. Due to the inherent complexity of sectors across environmental, social, and economic dimensions, there is no universally accepted representation of sectors. Each representation reflects a different worldview^46^. To provide a framework that can be operationalised and is transferable across nations, we based our sector classification on an original land-cover/land-use and international accounting classification adopted by most nations and global entities. Instead of focusing on impacts on sectors, one might also focus on systems of receptors, as discussed in the literature^63^. We opted for the internationally classified set of sectors, given our expectation that the framework can be applied with international and national accounting data across ecosystems and socio-economic sectors (see SEEA^28^).

Yet, this internationally based physical and mainly economic framing may mask cultural or nation-specific categorisations of indigenous peoples or marine ecosystems, amongst others. As the influence analysis in this paper (Step 2) focused on the service level, future work could group the service-level influences under different sector categories in ways that considers national differences. Future work is also required to better conceptualise the full range of services provided by each sector, especially in the context of ecosystems for which service allocation is complex^76^ and which often varies across spatial and temporal scales^30^. An understanding of the spatial and temporal changes in different services provided by ecosystems is crucial for sustainable ecosystem management^77,78^. Whilst ISIC provides a globally applicable overview of services linked to socio-economic sectors, no such international classification for ecosystems and the specific services these provide is available to date^30^. Future work can update the ecosystem categories and the services these provide accordingly, for example, using the SEEA classification being developed.

Importantly, the analysis in this paper does not consider income as a service provided by sectors, as at times adopted in previous studies^13^. We did not include income as a sector’s service, as it is provided by all sectors and can be indirectly linked to most SDG targets, thereby skewing the results to influence all targets. Future national-scale application might include the magnitude of income provided by each sector.

#### Climatic impact-driver definition/scope

We defined climatic impact-drivers based on IPCC’s AR5. We aimed to mitigate the absence of fire^79^ as a climatic impact-driver by including those influences whereby fires are exacerbated by droughts under the drying trend influences. We highlighted throughout our paper that droughts could be regarded as both an acute or chronic climatic impact-drivers. This differentiation does not influence our results (see Supplementary Data Tab 2.2).

#### SDG target definitions/scope

We utilised the SDG targets to provide a globally accepted framework of defining sustainable development, recognising that the targets are a political compromise rather than a scientific representation of all dimensions of sustainable development^70^. Our proposed framework is largely dependent on the wording used within each SDG target to determine direct sector-SDG influences. This focus on wording implies that the influence findings are affected by the often qualitatively delineated target descriptions that reflect the results of negotiations in intergovernmental contexts as opposed to science^46^. Yet, by focusing on the SDG target—rather than indicator— descriptions, we hope to address critiques of the use of indicators that are limited by globally available datasets^80^. Further, the target focus broadens the scope, applicability, and potential range of sectoral action (investments, policies, infrastructures) to influence the SDGs. Ambiguous direct or indirect sector-SDG influences were discussed within the author team, following previous literature in the field^4,10^.

#### Analysis

The majority of the analysis presented in this paper was based on evidence mapping of influences. Note that direct influences were based on content analysis and the indirect influences between utility infrastructure services and the SDG targets were taken from ref. ^10^ and updated based on new evidence published since 2019 (thereby yielding 58 more indirect influences). We acknowledge that for some sector-SDG influences or climate-SDG influences there might not be published evidence yet, therefore the absence of an influence in our manuscript does not necessarily imply the absence of a link. We based our manuscript on existing and published evidence to ensure findings are replicable and supported.

It is possible for existing literature to make erroneous inferences on influences, especially when based on grey literature. We aimed to mitigate this aspect by reviewing several grey literature studies for each influence and by discussing any potential issues or ambiguities with the authors of this paper, which span a range of disciplines and topical expertise (including geography, engineering, social science, ecosystems & biodiversity, infrastructure, climate risk analysis, SDG target mapping and climate adaptation). Yet, further research is required that uses the analysis on sector-SDG and climate-sector influences presented in this paper as a basis for more comprehensive systems analyses based on reinforcing or self-reinforcing loops, meta-analyses, or as inputs into systematic assessments. A systematic assessment could characterise the quality, quantity, and geographic focus of each influence. Additional data can help move from associative influences to quantitative causal inferences^22^, which helps result in more specific policy implication. Dynamic and machine learning-based literature evidence mappings^62^ can ensure that influence findings remain updated.

Sectors can have severe negative influences on SDG targets. Whilst we do not specifically assess negative influences or negative trade-offs amongst sectors, our analysis provides an indication of potential sector*-*SDG influences. These potential influences are often reversible, where a potential negative influence is—in the case of weak governance frameworks or corruption—also a positive influence. Future research should explicitly assess negative interdependent influences, for example by evaluating the impact of socio-economic sectors on ecosystem service provision. Previously established interaction scales that range from indivisible, reinforcing, to counteracting, or cancelling SDG target links^19,52^ could also be applied to sector-SDG, sector–sector, and climate-SDG influences to better identify the full range of positive and negative influences, including those for which more research is required.

#### Global application

With respect to our global application, the identification of near-term climate risk on sectors is based on the best available evidence of global sectoral near-term risk from the synthesis work reported in IPCC AR5^16^. As with any synthesis work, there remains some distance from the original studies and papers that reduce the researcher’s ability to judge the information accuracy^81^. Moreover, whilst IPCC AR5 considers a holistic set of climatic impact-drivers and multiple sectors, it is unclear to what extent a holistic set of sectors has been used in the sectoral risk assessment. Importantly, we acknowledge that public administration and its relevant governance services might not have been considered a sector by IPCC AR5’s risk ranking, largely under-estimating the range of SDG targets potentially affected. Future work can apply the framework to an updated overview of global sectoral risk that is explicit about the range of sectors it includes, such as for example IPCC’s AR6 or other global studies.

Risk is valued differently by different sectors and communities, making it challenging to compare across sectors^82^. Whilst the IPCC provided a first broad estimate of sectoral climate risk, it was not possible to: (i) compare the magnitude of climate risk across these sectors, (ii) identify the relative importance of climate stressors as compared to non-climate stressors, such as land-use or pollution, in affecting sectors and their service provision, (iii) account for systemic feedbacks amongst sectoral risks which may under- or over-represent climate risk. A more comprehensive approach should aim to compare climate risk amongst sectors, consider the amplification of risk across climate and non-climate stressors, and evaluate cascading impacts where possible.

The global application presented in this paper focused on a climate-first application of the framework. Whilst the discussion highlights how it is possible to work backwards from SDGs via sectors to reduce climate risk and deliver SDG co-benefits, further research can complement the analysis with a better understanding of: (i) how SDG targets guide sector action or sustainable service provision, and (ii) how different adaptation options can reduce risk from acute and chronic climatic impact-drivers and advance the SDG targets. This is particularly important in order to understand how leverage points not related to service provision—such as politics, people, culture, or technology— can help achieve SDG targets and contribute to climate adaptation. Further expansion of the framework that accounts for the climate mitigation potential of different sectors in reducing impacts of climatic impact-drivers could enable the framework to be used to identify climate-compatible (both low-carbon and resilient) pathways.

### Reporting summary

Further information on research design is available in the Nature Research Reporting Summary linked to this article.

## Supplementary information

Supplementary Information

Reporting Summary

Supplementary Data

## Data Availability

All data that support the findings of this study are available within the paper and its Supplementary Information (including all Supplementary Figures and Supplementary Tables). Supplementary Data are provided with this paper.
