# Author Correction: Targeting climate adaptation to safeguard and advance the Sustainable Development Goals

**DOI:** 10.1038/s41467-022-33518-z

**Published:** 2022-10-03

**Authors:** Lena I. Fuldauer, Scott Thacker, Robyn A. Haggis, Francesco Fuso-Nerini, Robert J. Nicholls, Jim W. Hall

**Affiliations:** 1 Environmental Change Institute, University of Oxford, South Parks Road, Oxford, OX1 3QY UK; 2 KTH Climate Action Centre & KTH Division of Energy Systems, KTH Royal Institute of Technology, SE-100 44 Stockholm, Sweden; 3 Tyndall Centre for Climate Change Research, University of East Anglia, Norwich, NR4 7TJ UK

**Keywords:** Climate-change impacts, Climate-change adaptation, Sustainability, Natural hazards, Climate-change impacts

**Correction to**: *Nature Communications* 10.1038/s41467-022-31202-w, published online 23 June 2022

The original version of this Article contained an error in the legend for Fig. 3, in which all colour-coded findings were incorrectly attributed as ‘Direct SDG influencers’, rather than only ’Unique’, ‘Cross-sectoral’, and ‘Substitute’. This has been corrected in both the PDF and HTML versions of the Article.

