# Supplementary material for: Targeting climate adaptation to safeguard and advance the Sustainable Development Goals
Source: Nat Commun. 2022 Jun 23;13:3579. doi: 10.1038/s41467-022-31202-w (PMC9226036; doi:10.1038/s41467-022-31202-w)
Supplement: Supplementary file 1 — Supplementary Information [file 41467_2022_31202_MOESM1_ESM.docx]

**Cover Page for Supplementary Information for the following manuscript:**

***Fuldauer, L.I., Thacker, S., Haggis, R.A., Fuso Nerini, F., Nicholls, R.J., Hall, J.W. Targeting climate adaptation to safeguard and advance the Sustainable Development Goals***

This Supplementary Information file contains Supplementary Figure 1 and Supplementary Tables 1-4, Supplementary Notes and References.

*Supplementary Figure 1:* Overview of how our proposed framework relates to hazard-, exposure-, and vulnerability-based adaptation. Grey shaded boxes refer to definitions of hazard, exposure, and vulnerability in relation to our proposed framework, based on IPCC AR5. Main box shows overview of how hazard-, exposure-, and vulnerability-based adaptation action relate to our framework of influences. Grey wordings indicate how mitigation and development action fit within the framework. Icon images courtesy of United Nations.

*Supplementary Table 1:* Overview of SDG targets and indicators integrated in National Adaptation Plans (NAPs) (https://www4.unfccc.int/sites/NAPC/Pages/national-adaptation-plans.aspx), status: February 2021. The symbol X indicates NAP does not mention SDGs, targets or indicators; XX describes NAP mentions SDGs, targets or indicators in the context of a single sector; XXX indicates NAP mentions SDGs, targets or indicators for all main sectors. See Supplementary Methods for overview of methods used to create Supplementary Table 1.

*Supplementary Table 2:* Overview of definitions of all key terms used within the manuscript.

*Supplementary Table 3:* Worked example for evidence of direct and indirect sector-SDG influences for two specific sectors: ‘wetland & peatland’ and ‘public administration’. The full range of evidence for the sector-climate influences is reported in Supplementary Information Tab 3.1.

*Supplementary Table 4:* Worked example for evidence of climate-sector influences for two specific sectors: ‘wetland & peatland’ and ‘public administration’. The full range of evidence for all sector-climate influences is reported in Supplementary Information Tab 3.2.

*Supplementary Notes*

*References*


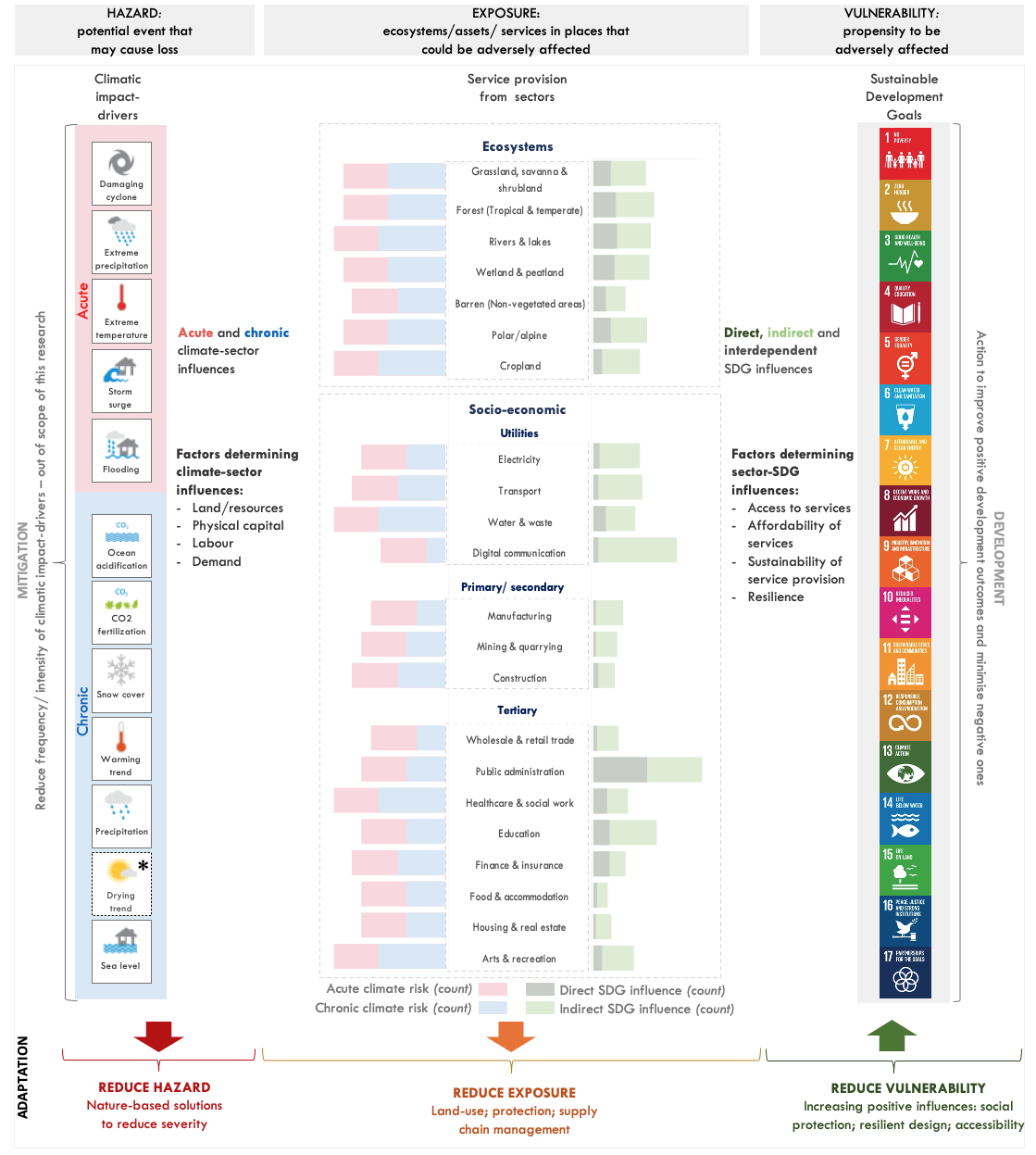


*Supplementary Figure 1: Overview of how our proposed framework relates to hazard-, exposure-, and vulnerability-based adaptation. Grey shaded boxes refer to definitions of hazard, exposure, and vulnerability in relation to our proposed framework, based on IPCC AR5. Main box shows overview of how hazard-, exposure-, and vulnerability-based adaptation action relate to our framework of influences. Grey wordings indicate how mitigation and development action fit within the framework.*

| Continent | Country | Year of NAP | 1. NAP mentions SDGs | 1. NAP mentions targets of SDGs | 3) NAP mentions SDG indicators |
| --- | --- | --- | --- | --- | --- |
| Africa and Middle East | Burkina Faso | 2015 |  |  |  |
|  | Cameroon | 2015 |  |  |  |
|  | Ethiopia | 2019 | X |  |  |
|  | Kenya | 2017 | X |  |  |
|  | Togo | 2018 | X |  |  |
|  | Sudan | 2016 | XX |  |  |
|  | Palestine | 2016 |  |  |  |
| Asia | Sri Lanka | 2016 | XXX | XXX |  |
| Pacific | Fiji | 2018 | XXX | XX |  |
|  | Kiribati | 2020 | XXX |  |  |
| Caribbean | St. Lucia | 2018 | XXX |  |  |
|  | St. Vincent and the Grenadines | 2019 | XXX | XXX | XXX |
|  | Grenada | 2019 | XXX | X | XXX |
| Latin America | Brazil | 2016 | XX |  |  |
|  | Colombia | 2018 |  |  |  |
|  | Chile | 2017 |  |  |  |
|  | Guatemala | 2019 | X |  |  |
|  | Paraguay | 2020 | XXX |  |  |
|  | Suriname | 2020 | XX |  |  |
|  | Uruguay | 2019 | XX |  |  |

*Supplementary Table 1: Overview of SDG targets and indicators integrated in National Adaptation Plans (NAPs) (https://www4.unfccc.int/sites/NAPC/Pages/national-adaptation-plans.aspx), status: February 2021. The symbol X indicates NAP does not mention SDGs, targets or indicators; XX describes NAP mentions SDGs, targets or indicators in the context of a single sector; XXX indicates NAP mentions SDGs, targets or indicators for all main sectors. See Supplementary Notes at the end of this file for overview of methods used to create Supplementary Table 1.*

| Term | | Definition (and examples) | Source (described in Supplementary Information (SI)) |
| --- | --- | --- | --- |
| Services provided by sector’s (ecosystem services: n=35; socio-economic services: n=32) | | Services provided by ecosystems:   - Regulating (flood protection or carbon sequestration), - Provisioning (food, water, transport, energy or medicines), - Supporting (habitat), and - Cultural services (heritage, recreational)   Services provided by socio-economic sectors:   - Infrastructure services such as electricity, transport, water - Primary/secondary services such as manufacturing, mining, etc. - Tertiary services such as law enforcement, education, etc. | USGS/ IUCN^1^ etc. (SI Tab 2.1)  ISIC Rev.4^2^  (SI Tab 2.1) |
| Direct SDG influence | | The SDG target is described directly in terms of the services that a sector provides. | Thacker et al. (2019)^3^  (SI Tab 3.1) |
| Inter-dependent SDG influence | Unique | The SDG target is described in terms of a single sector’s service only. This contribution cannot be substituted by the services of another sector. |  |
|  | Cross-sectoral | The SDG target is described in terms of multiple sectors’ services, where each sector’s service provides an independent contribution to the SDG target. |  |
|  | Substitutable | A substitutable SDG influence is identified when an SDG target is described in terms of sector’s service that can be substituted by a different sector. |  |
| Indirect SDG influence | | The SDG target is not described specifically in terms of the service that a sector provides, but published evidence indicates that improving the quality or quantity of the service provided by a sector can enhance the achievement of the target. |  |
| Climatic impact-driver (n=12) | | Acute climatic impact-drivers include: Cyclones; Extreme precipitation; Extreme temperature; Storm surge; Flooding and chronic climatic impact-drivers include: Ocean acidification; CO2 fertilization; Snow cover; Warming trend; Precipitation; Drying trend; Sea-level rise | IPCC AR5^4^  (SI Tab 2.2) |
| Climate influence | | An influence is identified if published evidence indicates that the climatic impact-driver affects the quantity or quality of services from the sector via effects on the supply factors (‘land/natural resources’, ‘physical capital’, or ‘labour’) or on directly quantifiable ‘demand’. | Adapted from Nerini et al. (2019)^5^  (SI Tab 3.2) |
| Near-term risk | | *Near-term risk* refers to key sectoral risk of climatic impact-drivers and is defined based on a function of hazard (high probability/large magnitude), persistent exposure and vulnerability in the 2030s (aligned with the SDG timeline). A sector is considered at high near-term global risk (according to IPCC AR5), if sector or its services, as worded in SI Tab 2.1 is at high/very high risk with current adaptation levels and high confidence. | IPCC AR5 Table TS4^4^  IPCC 1.5^6^ |

*Supplementary Table 2: Overview of definitions of all key terms used within the manuscript. See references at the end of this document.*

| SDG Target description | | Ecosystems | | Socio-economic | |
| --- | --- | --- | --- | --- | --- |
|  |  | Wetland & peatland | | Public administration | |
|  |  | Services:  • Provisioning: Food (e.g. fish, wild rice), freshwater, natural habitat, natural resources, seeds, sanitation  • Regulating: Natural flood protection creating resilience; carbon sequestration; purification of water  • Cultural services: spiritual and cultural significance; recreation; education; green space; health and wellbeing | | Services:  • Public administration and defence;  • Creation/ implementations of programmes and policies  • Governance  • Social protection and compulsory social security (includes services such as administration of the state and the economic and social policy of the community)  • Foreign affairs; defence activities;  • Administration and regulation of the activities of providing health care, education, portable water, cultural services and other social services and access to basic services | |
| 1.1 | By 2030, eradicate extreme poverty for all people everywhere, currently measured as people living on less than $1.25 a day |  | Indirect influence: nature, including wetlands, provide import contributions to livelihoods for the large number of people still living in extreme poverty (1). |  | Indirect influence: improving the quality / quantity of services delivered by the public administration sector can enhance this target, through the role of policy and regulation in combatting extreme poverty (1). |
| 1.2 | By 2030, reduce at least by half the proportion of men, women and children of all ages living in poverty in all its dimensions according to national definitions |  | Indirect influence: improving the quantity / quality of services provided by this sector can enhance achievement of this target through various ecosystem services, such as the provisioning of water and fuel, and their ability to support income generation that helps reduce the proportion of the population living in poverty (2) (3). |  | Indirect influence: Poverty is characterized by severe deprivation of 'basic human needs', which include access to basic services. Therefore, enhancing the quality / quantity of services provided by this sector can impact this target through administration and regulation of basic services (2) (3). |
| 1.3 | Implement nationally appropriate social protection systems and measures for all, including floors, and by 2030 achieve substantial coverage of the poor and the vulnerable |  |  | Direct influence: public administration provides social protection and security activities (funding and administration of government-provided social security programmes) |  |
| 1.4 | By 2030, ensure that all men and women, in particular the poor and the vulnerable, have equal rights to economic resources, as well as access to basic services, ownership and control over land and other forms of property, inheritance, natural resources, appropriate new technology and financial services, including microfinance | Direct influence: provision of natural resources |  | Direct influence: enactment and judicial interpretation of laws and their pursuant regulation. Services include administration of programmes which deliver access to basic services, including health, education. |  |
| 1.5 | By 2030, build the resilience of the poor and those in vulnerable situations and reduce their exposure and vulnerability to climate-related extreme events and other economic, social and environmental shocks and disasters | Direct influence: natural flood protection creating resilience |  |  | Indirect influence: improving the quality / quantity of services delivered by the public administration sector can enhance the achievement of this target through the implementation of policies and regulations that address climate change and build resilience (4) (5). |
| References: | |  | (1) IPBES., Global assessment report on biodiversity and ecosystem services of the Intergovernmental Science-Policy Platform on Biodiversity and Ecosystem Services. E. S. Brondizio, J. Settele, S. Díaz, and H. T. Ngo (editors). IPBES secretariat, Bonn, Germany. (2019). Chapter 3, p.100.  (2) Verma, M., & Negandhi, D., Valuing ecosystem services of wetlands – a tool for effective policy formulation and poverty alleviation. Hydrological Sciences Journal, 56, 8, 1622-1639 (2011). Available at: <https://www.tandfonline.com/doi/full/10.1080/02626667.2011.631494>  (3) Kumar, R., Horwitz, P., Milton, R., Sellamuttu, S. S., Buckton, S. T., Davidson, N. C., Pattnaik, A. K., Zavagli, M., & Baker, C., Assessing wetland ecosystem services and poverty interlinkages: a general framework and case study. Hydrological Sciences Journal, 56, 1602-1621 (2011). Available at: |  | (1) Bicaba, Z., Brixiova, Z., Ncube, M. Eliminating extreme poverty in Africa: The role of policies and global governance. International Growth Centre (2016). Available at:  https://www.theigc.org/blog/eliminating-extreme-poverty-in-africa-the-role-of-policies-and-global-governance/. (Accessed: 20th March 2020)  (2) United Nations. Report of the world summit for social development. (United Nations, Copenhagen, 1995).  (3) United Nations. International Standard Industrial Classification of all Economic Activities (ISIC), Rev. 4. UN Department of economic and Social Affairs: statistics division (2008). Available at: <https://unstats.un.org/unsd/publication/seriesM/seriesm_4rev4e.pdf>  (4) Science Direct. Climate Policy. Available at: <https://www.sciencedirect.com/topics/social-sciences/climate-policy>  (5) Vallejo, L., & Mullan, M., Climate-resilient infrastructure: getting the policies right. (2017). Available at: https://www.greengrowthknowledge.org/resource/climate-resilient-infrastructure-getting-policies-right |

*Supplementary Table 3: Worked example for evidence of direct and indirect sector-SDG influences for two specific sectors: ‘wetland & peatland’ and ‘public administration’. The full range of evidence for the sector-climate influences is reported in Supplementary Information Tab 3.1.*

|  |  | Ecosystems | Socio-economic |
| --- | --- | --- | --- |
|  | CLIMATIC IMPACT- DRIVER | Wetland & peatland | Public administration |
| Acute climatic impact-driver | Extreme precipitation | [Land] Given the proximity of fishing and aquaculture sites to oceans, seas, and riparian environments, extreme events can be expected to have impacts on fisheries and aquaculture with those located in low-lying areas at particular risk (Porter et al., 2014). | [Capital] Extreme precipitation impacting critical assets (IPCC, 2014), affecting the functioning of public administration assets. |
|  | Extreme temperature | [Land] Heat waves lead to widespread seagrass mortality, as documented for Zostera species in the Atlantic (Reusch et al., 2005) and Posidonia meadows in the Mediterranean Sea (Marbà and Duarte, 2010) and Australia (Rasheed and Unsworth, 2011; high confidence) (as cited in Wong et al., 2014), which can influence the ability of wetlands to sustain biodiversity and habitat.  [Land] Extremely hot days exceeding a certain duration can determine species range and abundance and potentially facilitate spread of pests and pathogen outbreaks (Zimmermann et al., 2009; Settele et al., 2014). | [Capital] Heat stress impacting critical assets (IPCC, 2014), affecting the functioning of public administration assets. |
| Chronic climatic impact-driver | Sea level rise | [Land] Relative sea level rise can result in wetland loss and change (Nicholls et al., 2014) (as cited in Wong et al., 2014).  [Land] Vegetated coastal habitats are declining globally (Duarte et al., 2005), rendering shorelines more vulnerable to erosion due to increased sea level rise and increased wave action (e.g., Alongi, 2008) and leading to the loss of carbon stored in sediments (as cited in Wong et al., 2014), reducing the ability of wetlands to maintain natural cycles. | [Capital] Coastal critical infrastructure, including public administration departments housing critical information, exposed to coastal risks due to sea level rise (IPCC, 2014). Exposed critical infrastructure reduces the ability of the sector to perform its basic functions. |
|  | Warming trend | (+ -) [Land] Ocean warming is leading to range shifts in vegetated coastal habitats. Accordingly, migration of the isotherm with climate change (Burrows et al., 2011) should lead to a poleward expansion of mangrove forests, as observed in the Gulf of Mexico (Perry and Mendelsohn, 2009; Comeaux et al., 2011; Raabe et al., 2012) and New Zealand (Stokes et al., 2010), leading to increased sediment accretion (medium confidence) (as cited in Wong et al., 2014).   (+ -) [Land] High confidence in observations of increasing abundance of fish species in the northern extent of their ranges while decreases in abundance have occurred in the southern part. These trends will have mixed implications for fisheries and aquaculture with some commercial species negatively and others positively affected (Cook and Heath, 2005) (as cited in Porter et al., 2014). | [Demand] Deviation from normal temperature was found to be correlated to increase the risk of conflict (Hsiang et al, 2013), influencing the demand for public administration and defence. |
|  | References | Porter, J. R. et al. Food security and food production systems. in Climate Change 2014: Impacts, Adaptation, and Vulnerability. Part A: Global and Sectoral Aspects. Contribution of Working Group II to the Fifth Assessment Report of the Intergovernmental Panel on Climate Change (IPCC, 2014).  Wong, P. P. et al. Coastal systems and low-lying areas. in Climate Change 2014: Impacts, Adaptation, and Vulnerability. Part A: Global and Sectoral Aspects. Contribution of Working Group II to the Fifth Assessment Report of the Intergovernmental Panel of Climate Change (IPCC, 2014).  Settele, J. et al. Terrestrial and Inland water systems. in Climate Change 2014: Impacts, Adaptation, and Vulnerability. Part A: Global and Sectoral Aspects. Contribution of Working Group II to the Fifth Assessment Report of the Intergovernmental Panel on Climate Change (IPCC, 2015). | IPCC 2014. Climate Change 2014: Synthesis Report. Contribution of Working Groups I, II and III to the Fifth Assessment Report of the Intergovernmental Panel on Climate Change [Core Writing Team, R.K. Pachauri and L.A. Meyer (eds.)]. (IPCC, 2014).  Hsiang, S. M. Temperatures and cyclones strongly associated with economic production in the Caribbean and Central America. Proc. Natl. Acad. Sci. U. S. A. 107, 15367–15372 (2010). |

*Supplementary Table 4: Worked example for evidence of climate-sector influences for two specific sectors: ‘wetland & peatland’ and ‘public administration’. The full range of evidence for all sector-climate influences is reported in Supplementary Information Tab 3.2.*

Supplementary Notes

Overview of methods for Supplementary Table 1. We searched each NAP (https://www4.unfccc.int/sites/NAPC/Pages/national-adaptation-plans.aspx) using the following search terms (based on the status of NAPs in January 2021; SI refers to Supplementary Information Tab):

1: NAP mentions SDGs

- ‘some mention’: SDGs, Sustainable Development Goals, Agenda 2030
- ‘clearly mentioned in the context of a single sector’: SDGs, Sustainable Development Goals, Agenda 2030 & one sector: either agriculture, forest, water, energy, (other sectors as specific in (Supplementary Information Tab (SI) 2.1)
- ‘clearly mentioned for all main sectors’: SDGs, Sustainable Development Goals, Agenda 2030 & more than one sector name, cross-cutting, cross-sectoral

2: NAP mentions the targets of the SDGs

- ‘some mention’: SDG targets, development targets, sustainable development targets, Agenda 2030 targets
- ‘clearly mentioned in the context of a single sector’: SDGs, Sustainable Development Goals, Agenda 2030 & one sector: either agriculture, forest, water, energy, (other sectors as specific in SI 2.1)
- ‘clearly mentioned for all main sectors’: SDGs, Sustainable Development Goals, Agenda 2030 & more than one sector name: agriculture, forest, water, energy, (other sectors as specific in SI 2.1), cross-cutting, cross-sectoral

3: NAP mentions SDG indicators

- ‘some mention’: SDG indicators, SDG targets, development targets, sustainable development targets, Agenda 2030 targets
- ‘clearly mentioned in the context of a single sector’: SDG indicators, SDG targets, development targets, sustainable development targets, Agenda 2030 targets & one sector: either agriculture, forest, water, energy, (other sectors as specific in SI 2.1)

‘clearly mentioned for all main sectors’: SDGs, Sustainable Development Goals, Agenda 2030 & more than one sector name: agriculture, forest, water, energy, (other sectors as specific in SI 2.1), cross-cutting, cross-sectoral

References for Supplementary Table 2:

1. Anderson, J. R., Hardy, E. E., Roach, J. T. & Witmer, R. E. *A Land Use and Land Cover Classification System for Use with Remote Sensor Data*. *Geological Survey Professional Paper 964* (1976).

2. UN. *International Standard Industrial Classification of All Economic Activities (ISIC), Revision 4*. (United Nations, 2008).

3. Thacker, S. *et al.* Infrastructure for sustainable development. *Nat. Sustain.* **2**, 324–331 (2019).

4. Field, C. B. *et al.* Technical summary. in *Climate Change 2014: Impacts, Adaptation, and Vulnerability. Part A: Global and Sectoral Aspects. Contribution of Working Group II to the Fifth Assessment Report of the Intergovernmental Panel on Climate Change* (IPCC, 2014).

5. Fuso Nerini, F. *et al.* Connecting climate action with other Sustainable Development Goals. *Nat. Sustain.* **2**, 674–680 (2019).

6. Masson-Delmotte, V. *et al.* *Global warming of 1.5°C. An IPCC Special Report on the impacts of global warming of 1.5°C above pre-industrial levels and related global greenhouse gas emission pathways, in the context of strengthening the global response to the threat of climate change,*. *Report of the Intergovernmental Panel on Climate Change* (2018).
